# Diagnostic performance of the loop-mediated isothermal amplification (LAMP) based illumigene^®^ malaria assay in a non-endemic region

**DOI:** 10.1186/s12936-017-2065-8

**Published:** 2017-10-17

**Authors:** Anne-Sophie De Koninck, Lieselotte Cnops, Mattias Hofmans, Jan Jacobs, Dorien Van den Bossche, Jan Philippé

**Affiliations:** 1 Department of Laboratory Medicine, Ghent University Hospital (GUH), De Pintelaan 185, 9000 Ghent, Belgium; 2 Institute of Tropical Medicine (ITM) Antwerp, Nationalestraat 155, 2000 Antwerp, Belgium

**Keywords:** Illumigene malaria assay, LAMP assay, Plasmodium, Loop-mediated isothermal amplification, Malaria

## Abstract

**Background:**

Light microscopy and antigen-based rapid diagnostic tests are the primary diagnostic tools for detecting malaria, although being labour-intensive and frequently challenged by lack of personnel’s experience and low levels of parasite density. The latter being especially important in non-endemic settings. Novel molecular techniques aim to overcome this drawback. The objective of this study was to assess the diagnostic performance of the illumigene malaria assay^®^ (Meridian Bioscience) compared to microscopy, RDT and real-time PCR. This loop-mediated isothermal amplification (LAMP) assay is a qualitative in vitro diagnostic test for the direct detection of *Plasmodium* spp. DNA in human venous whole blood samples.

**Methods:**

The illumigene assay was assessed on a retrospective panel of stored blood samples (n = 103) from returned travellers and external quality control samples (n = 12). Additionally the assay was prospectively assessed on 30 fresh routine samples with a request for malaria diagnosis. The illumigene assay was compared to microscopy, RDT and *Plasmodium* species specific real-time PCR.

**Results:**

In the retrospective evaluation, the illumigene assay showed 100% agreement with the real-time PCR, RDT and microscopy yielding a sensitivity and specificity of 100% (95% CI 95.1–100% and 89.7–100%, respectively). Seven samples from patients recently treated for *Plasmodium falciparum* infection that were RDT positive and microscopy negative yielded positive test results. The performance of the illumigene assay equals that of microscopy combined with RDT in the prospective panel with three false negative RDT results and one false negative microscopy result. Excellent concordance with PCR was observed. The limit of detection of the assay approached 0.5 parasites/µL for both *P. falciparum* and *Plasmodium vivax*.

**Conclusion:**

In non-endemic regions where the diagnostic process for malaria infections is questioned by lack of experience and low levels of parasite densities, the illumigene assay can be of value. Due to its high sensitivity, the LAMP assay may be considered as primary diagnostic test. The results of this study indicate that negative screen results do not need further confirmation. However, before implementation, this approach needs to be confirmed in larger, prospective studies. A shortcoming of this assay is that no species identification nor determination of parasite density are possible.

## Background

Imported malaria infections in non-endemic regions remain common due to increasing travel to endemic malaria areas and population movements [1–4]. Yearly, according to the World Health Organization (WHO) [5, 6], approximately 10,000 cases of imported malaria are reported, but the actual number may be as high as 30,000. Rapid and accurate diagnosis of infections with species identification and determination of parasite density is essential to optimize treatment and reduce mortality [7–9]. However, diagnosis of imported cases can be challenging due to infrequent encounters [3, 10], leading to difficulties in diagnosis [11], misdiagnosis and delays in treatment.

Light microscopy and quality assured antigen-based rapid diagnostic tests (RDTs) are the primary diagnostic tools for confirmation and management of cases of suspected clinical malaria in a non-endemic setting. Microscopic examination of Giemsa-stained blood slides (thin and thick films) by trained and experienced staff has been the standard for malaria diagnosis for nearly a century [1, 12]. However this technique is labour-intensive, time consuming and challenged by a high limit of detection (LoD). The latter, in ideal conditions estimated to be close to 50 parasites/µL, strongly depends on the quality of the slide and the training level of the microscopist [13]. Furthermore, personnel’s lack of practice and proficiency may account for delays and errors in diagnosis [11]. No alternative method has been accepted to replace this gold standard method yet [1, 12]. RDTs were introduced to overcome some of these drawbacks. Most RDTs currently used are based on immunochromatographic techniques which detect histidine-rich protein 2 (HRP-2), a protein specific to *Plasmodium falciparum*, and pan-*Plasmodium* parasite lactate dehydrogenase (pan-pLDH) or aldolase, enzymes common to all *Plasmodium* species [7]. Because RDTs are fast, easy to use, generally have good sensitivity for *P. falciparum* and have a relatively low cost, they are a valuable adjunct to (but not a replacement for) microscopy for the diagnosis of malaria in the returned traveller [2, 6]. The sensitivity for *P. falciparum* is excellent at parasite densities > 100/µL (median 94.3%, range 77.4–98.1%), but significantly lower at densities below 100/µL (median 74.1%, range 9.1–88.5%). Of note, the sensitivity for non-falciparum species varies between different RDTs used (*Plasmodium vivax*: 66.0–88.0%; *Plasmodium ovale*: 5.5–86.7%; *P. malariae*: 21.4–45.2%), with a marked decline in sensitivity at parasite densities below 500/µL [6, 14].

Molecular techniques have drawn attention because they can overcome the most important disadvantages of RDTs such as decreasing sensitivity at low parasite density and limited capacity for identification of non-*P. falciparum* and mixed-species infections [6, 15–17]. Nucleic acid amplification techniques (NAATs) are several orders of magnitude more sensitive than microscopy and RDTs [2]. Parasite densities in the case of *P. falciparum* in returned travellers may be below the RDT threshold and below the (non-expert and expert) microscopy threshold [6, 18]. Previously published data suggested that approximately 10% of patients with *P. falciparum* infection presented with parasite density below 100/µL at the Institute of Tropical Medicine (ITM), Belgium [6]. Moreover, non-immune patients may develop clinical disease at very low parasite densities [18].

Polymerase chain reaction (PCR) techniques used to diagnose malaria infections include single-step, nested, multiplex and quantitative, conventional or real-time PCR formats. Other NAATs do not require thermal cyclers, the most common being loop-mediated isothermal amplification (LAMP) and nucleic acid sequence-based amplification. The sensitivity of LAMP method is reported to approach that of nested PCR [19] and unlike PCR, samples for LAMP do not require extensive preparation [20].

The main objective of our study was to assess the diagnostic performance of the illumigene Malaria DNA Amplification assay^®^ (Meridian Bioscience, Inc., Cincinnati OH, USA), compared to microscopy, RDT and real-time PCR for *Plasmodium* spp. detection.

## Methods

### Study design

In this study, the diagnostic performance of the illumigene assay was assessed. In the retrospective part of the study, the assay was assessed against a selection of stored samples (n = 103) obtained from international travellers, collected from June 2015 to June 2016 and external quality controls (EQC) (n = 12). The malaria RDT, microscopic evaluation and real-time PCR [1] were carried out at the reference laboratory of the Institute of Tropical Medicine (ITM) Antwerp, Belgium. The illumigene assay was performed retrospectively at the clinical laboratory of the Ghent University Hospital (GUH).

Furthermore, the illumigene assay was prospectively assessed between July 2016 and September 2016 on 30 samples obtained from patients suspected to have malaria infection at the clinical laboratory of the Ghent University Hospital (GUH). The malaria RDT, microscopic evaluation and illumigene assay were completed at the GUH. Thereafter, all positive and discordant samples were sent to ITM for species identification and determination of parasite density. This study complied with the standards for the reporting of diagnostic accuracy studies (STARD).

### Clinical samples (retrospective and prospective)

A panel of stored EDTA-anticoagulated venous whole blood diagnostic samples (n = 103) and EQC samples (n = 12) was analysed. Blood samples were obtained from returned travellers who presented at the outpatient clinic of the ITM or were submitted by diagnostic laboratories for malaria confirmation to the Central Laboratory of Clinical Biology of ITM (the Belgian reference laboratory for *Plasmodium*, accredited according to ISO15189:2012). This panel contained 66 *Plasmodium* positive samples covering the different *Plasmodium* species (27 *P. falciparum*, 14 *P. vivax*, 13 *P. ovale*, 12 *P. malariae*) as identified by real-time PCR (Cnops et al. [1]). Parasite densities were quantified microscopically on thick film by counting the asexual parasites (no gametocytes) per 200 white blood cells and converting this count to asexual parasites per microlitre. The parasite densities of the samples varied between 1–372,117 asexual parasites/µL (see Table 1 for more details). Also, 30 *Plasmodium* negative specimens were included. These samples tested *Plasmodium*-negative through standard microscopy, RDT and real-time PCR. This negative panel included 5 samples to check for cross-reactivity (cfr. infra). Additionally 7 samples from patients who already received treatment for malaria infection were retrospectively tested (n = 7/103). The EQC samples (from UK NEQAS) covered the 5 different species of *Plasmodium* (n = 12, incl. 1 *Plasmodium knowlesi*) (Table 1).

**Table 1:** Shows malaria diagnosis for 115 retrospective samples by the illumigene malaria assay in comparison with standard microscopy, RDT and PCR

	Reference method (RDT, microscopy, PCR)		Illumigene malaria assay		
	Results (n)	Range parasite density (asexual parasites/µL)	Results (n)	Performance	
Total	108		108		
Positive	74		74	Sensitivity (%) (95% CI)	100% (95.1–100%)
Negative	34		34	Specificity (%) (95% CI)	100% (89.7–100%)
Clinical samples	96				
*Pf*	27	(1–372,117)	–*		
*Pv*	14	(169–18,777)	–*		
*Pm*	13	(106–9244)	–*		
*Po*	12	(31–6461)	–*		
Mixed	0				
Negative	30				
External quality controls	12				
*Pf*	1	5	–*		
*Pv*	1	5	–*		
*Pm*	2	5	–*		
*Po*	3	20	–*		
*Pk*	1	10,000			
Negative	4				

The retrospective panel consisted of 103 clinical samples (96 diagnostic samples and 7 samples from patients who already received treatment for malaria infection) and 12 external quality controls. These 7 samples from patients who already received treatment (115 − 7 = 108) were excluded from the calculation of both sensitivity and specificity. Parasite density as quantified by microscopy on blood smear

RDT, rapid diagnostic test; PCR, four-primer real-time PCR; *Pf*, *Plasmodium falciparum*; *Pm*, *Plasmodium malariae*; *Po*, *Plasmodium ovale*; *Pv*, *Plasmodium vivax*; *Pk*, *Plasmodium knowlesi*; n, number of samples; –*, the illumigene malaria assay does not distinguish between *Plasmodium* species

All samples were stored at − 80 °C up to 1 year and had not been thawed before analysis. Performance of the illumigene assay on samples stored for more than 1 month up to 1 year at − 80 °C (i.e. maximal storage time according to the manufacturer, Meridian Bioscience [21]) was evaluated on the first set of 20 *Plasmodium* positive samples.

The prospective panel consisted of thirty freshly drawn venous EDTA-anticoagulated whole blood samples consecutively collected from patients with clinical suspicion of malaria infection, and submitted to the laboratory of the GUH. These samples were stored for a maximum of 1 week at 4 °C, meeting the specifications of the manufacturer. The illumigene assay was performed on de-identified residual samples by laboratory technicians blinded from RDT, microscopy and PCR test results.

### Diagnostic test methods

The illumigene assay is a qualitative in vitro diagnostic LAMP test for the direct detection of *Plasmodium* spp. DNA in human venous EDTA whole blood samples (50 µL). The assay targets a region of the *Plasmodium* genome that is conserved across *P. falciparum*, *P. vivax*, *P. ovale*, *P. malariae* and *P. knowlesi*, i.e. a 214 bp sequence of the *Plasmodium* spp. mitochondrial DNA noncoding region. The assay does not distinguish between the different *Plasmodium* species [21]. The price per test device (in euro, excl. VAT) is estimated 28€.

The assay uses a simple filtration workflow (SMP-PREP™) to extract DNA from EDTA-anticoagulated whole blood, a procedure relying on chemical lysis which produces amplifiable DNA within 10 min [20, 22]. Fifty microlitres of the whole blood sample is added to a collection tube containing 320 µL of illumigene lysis buffer and is thoroughly vortexed. After a 2 min incubation at room temperature, 50 µL of the lysate is added to a simple sample device with filter (SMP PREP IV) containing 900 µL of reaction buffer. After inverting five times, 5–10 drops are gently squeezed from this device into a clean eppendorf tube. 50 µL of this eluate was added to both the test and control chamber of the illumigene test device [20, 22]. This test device consists of a TEST tube containing primers targeting the genus *Plasmodium* and a CONTROL tube with primers detecting the housekeeping human gene acting as an amplification control. The LAMP assay was performed using the Illumipro-10™ Incubator/Reader, which is capable of testing maximal 10 samples in a single run. The change in turbidity associated with LAMP amplification, due to the magnesium-pyrophosphate build-up as a by-product, is visually detected by the reader and a qualitative result is determined (positive, negative or invalid) [20].

Each illumigene test device contains an internal control that controls for amplification inhibition, assay reagents, DNA preparation, and sample processing effectiveness [23]. It is however recommended by the manufacturer that the reactivity of each new lot and each new shipment of illumigene test kits be verified on receipt and before use. External control tests should be performed in accordance with appropriate federal, state and local guidelines. Illumigene external control reagent is supplied separately; alternatively, previously characterized clinical or contrived *Plasmodium* spp. positive blood samples can be used as an external positive control. A qualified negative human whole blood sample may be used as an external negative control. The test kit should not be used in patient testing if the external controls do not produce the correct results [23].

### Reference method

The retrospective panel of clinical samples was tested at the reference laboratory of ITM using microscopy (Giemsa stained thin and thick film) and multiple RDTs: the SD FK60 Malaria Ag Pf/pan RDT-test (Alere, Waltham, Massachusetts, USA), which detects *P. falciparum*-specific HRP-2 and pan-pLDH [17]; the CareStart^TM^ Malaria pLDH 3 line test (AccessBio, Somerset, New Jersey, USA), which detects *P. falciparum*-specific parasite lactate dehydrogenase (Pf-pLDH) and pan-pLDH [15]; and the SD FK80 Malaria Ag *P.f*/*P.v* test in case of *P. vivax* suspicion as it detects *P. vivax*-specific pLDH [24]. All retrospectively analysed samples (positive and negative) were confirmed with real-time PCR [1]. Analytical sensitivity of this real-time PCR is 0.02 asexual parasites/µL for *Pf*/*Pv*, 0.004 for *P. ovale* and 0.006 for *P. malariae* [1].

The prospectively collected samples were compared with standard-of-care testing at the clinical laboratory of GUH. Routine testing consisted of RDT analysis (BinaxNOW^®^ malaria test, Alere), followed by microscopic evaluation of Giemsa-stained thin and thick film by experienced staff. All positive and discordant samples were sent to reference laboratory of the ITM for species identification and assessment of parasite density. Negative samples with both microscopy and RDT were not sent to the reference laboratory for confirmation.

### Statistical analysis

Sensitivity and specificity with 95% confidence intervals (CI) of the illumigene assay were determined using the real-time PCR as the reference test. All statistical analysis were performed with MedCalc software version 11.6.1.0 (MedCalc Software, Ostend, Belgium).

### Analytical sensitivity

The LoD of the illumigene assay is claimed by the manufacturer to be 2 parasites/µL for *P. falciparum* and 0.125/µL for *P. vivax* [21]. In order to verify this LoD, blood samples from a patient with *P. falciparum* (parasite density = 6272/µL) and *P. vivax* (parasite density = 7285/µL) were diluted with uninfected blood from a healthy donor of the same blood group. Tenfold serial dilution was made to obtain a final parasite density of approximately 0.5 parasite/µL. Dilution and analysis of samples were performed in duplicate.

### Cross-reactivity

To test the illumigene assay for cross-reactivity, 3 clinical samples with a PCR-confirmed infection with resp. *Leishmania infantum*, *Trypanosoma brucei rhodesiense*, and *Schistosoma mansoni* and 2 samples with a high concentration of rheumatoid factor were tested.

## Results

### Diagnostic method comparison

The retrospective analysis of clinical samples (n = 103) and EQC samples (n = 12) showed complete agreement between the illumigene assay and the reference methods (RDT, microscopy and PCR) (Table 1). No invalid LAMP results were observed. In a first set of 20 *Plasmodium* positive samples stored at − 80 °C for up to 1 year, all samples showed a positive result with the illumigene assay, justifying the evaluation of the assays’ performance on samples stored more than 1 month up to 1 year at − 80 °C. The illumigene assay demonstrated a sensitivity of 100% (95% CI 95.1–100%) and specificity of 100% (95% CI 89.7–100%) [samples from patients already treated for malaria infection not included (7/103)]. These additional tested samples from patients recently treated for *P. falciparum* infection (n = 7) also yielded positive test results and showed agreement with real-time PCR [1]. The median age of the patients was 36 (range 4–79) with 12 (11.7%) < 15 years old. The male/female ratio was 72:31. The country of travel was known for 93 (90.3%) patients. Most returned from Africa (80.7%), followed by Asia (14.0%) and South-America (5.3%). The top three countries of destinations were Democratic Republic Congo (n = 17), Cameroon (n = 8), Côte d’Ivoire and Ghana (n = 6).

During the prospective validation, 30 consecutive samples were tested: 18 (60%) were identified as negative with the illumigene assay and 12 (40%) as positive (Table 2). The median age of the patients was 36 (range 8–68) with 4 (11.7%) ≤ 15 years old. The male/female ratio was 20:10. The country of travel was known for 29/30 (96.7%) of travellers. Most returned from Africa (58.6%), followed by Asia (34.5%) and South-America (6.9%). In 25/30 samples the status of malaria prophylaxis was known. Malaria prophylaxis was taken in 20% (5/25). No invalid results were observed. The negative illumigene assay results were in complete agreement with RDT and microscopy (n = 18, 100%), whereas the positive results showed excellent agreement with real-time PCR results for all samples tested (11/11, 100%). Species identification with PCR showed 4 *P. falciparum*, 3 *P. vivax* and 2 *P. ovale* single infections and 2 mixed infections (2 *P. falciparum* + *P. ovale*). Confirmation with PCR of 1 sample was not available (sample 12).

**Table 2:** Shows malaria diagnosis for 30 whole blood samples with the illumigene malaria assay in comparison with standard microscopy, RDT and PCR

Sample	Illumigene	RDT	Microscopy	PCR		Parasite density (parasites/µL)
				Result	Species	
1	Pos	Pos	Pos	Pos	*Pf* + *Po*	1,027,798
2	Pos	Pos	Pos	Pos	*Pf*	292,432
3	Pos	Pos	Pos	Pos	*Pv*	68,129
4	Pos	Pos	Pos	Pos	*Pf*	27,731
5	Pos	Pos	Pos	Pos	*Pv*	7285
6	Pos	Pos	Pos	Pos	*Pf*	5917
7	Pos	Pos	Pos	Pos	*Pf* + *Po*	98
8	Pos	Pos (*)	Pos	Pos	*Po*	1496
9	Pos	***Neg***	Pos	Pos	*Po*	59
10	Pos	***Neg***	Pos	Pos	*Pv*	340
11	Pos	***Neg***	Pos	Pos	*Pf*	415
12	Pos	Pos	***Neg***	− (#)	–	–
13	Neg	Neg	Neg	/		
14	Neg	Neg	Neg	/		
15	Neg	Neg	Neg	/		
16	Neg	Neg	Neg	/		
17	Neg	Neg	Neg	/		
18	Neg	Neg	Neg	/		
19	Neg	Neg	Neg	/		
20	Neg	Neg	Neg	/		
21	Neg	Neg	Neg	/		
22	Neg	Neg	Neg	/		
23	Neg	Neg	Neg	/		
24	Neg	Neg	Neg	/		
25	Neg	Neg	Neg	/		
26	Neg	Neg	Neg	/		
27	Neg	Neg	Neg	/		
28	Neg	Neg	Neg	/		
29	Neg	Neg	Neg	/		
30	Neg	Neg	Neg	/		

Parasite density as quantified by microscopy on blood smear

RDT, rapid diagnostic test; PCR, four-primer real-time PCR; *Pf*, *Plasmodium falciparum*; *Pm*, *Plasmodium malariae*; *Po*, *Plasmodium ovale*; *Pv*, *Plasmodium vivax*; Pos, positive; Neg, negative. –, missing data; (#), negative result with real-time PCR performed on thin blood smear, possibly due to inferior sample quality; (*), difficult to interpret (weak positive result); /, triple negative results (i.e. negative with RDT, microscopy and illumigene malaria assay) were not sent to the ITM for confirmation with real-time PCR

The illumigene assay showed 1 (1/30; 3.3%) discordant result compared to microscopy (sample 12: positive illumigene test result, positive RDT result, but negative microscopic result). No left-over of whole blood was available for real-time PCR testing. PCR on a thin blood smear of this sample could not confirm the positive result of the illumigene assay. As compared with the RDT used (BinaxNOW), three additional positive samples (sample 9: 59 *P. ovale* parasites/µL; sample 10: 340 *P. vivax* parasites/µL and sample 11: 415 *P. falciparum* parasites/µL) were detected with the illumigene assay (agreement in 27/30 samples, 90%). These three samples were confirmed positive with the reference method at ITM (microscopy, RDT and PCR). In contrast to the BinaxNOW, other RDTs used at the ITM showed positive results for these samples (sample 9: positive with Carestart panLDH and negative with SD; sample 10: positive with panLDH, Carestart panLDH and SD Pv; sample 11: positive with SD HRP-2 and Carestart PfLDH). Sample 11 was obtained from a patient, with a history of multiple (n = 6) malaria infections, who traveled to Sierra Leone for 4 months without appropriate malaria prophylaxis and returned 3 days prior to sampling. In all 3 cases the BinaxNOW RDT was repeated and showed consistently negative results. Furthermore, another sample was initially wrongly interpreted as RDT negative by a trained laboratory technician, but showed a faint positive result upon second assessment of the RDT afterwards. This sample revealed positive result with the illumigene assay (sample 8).

### Analytical sensitivity

Positive test results were obtained for all concentrations tested with *P. falciparum* (i.e. 6272 parasites/µL, 1045/µL, 104.5/µL, 10.5/µL, 2/µL, 1/µL and 0.5/µL) and *P. vivax* (i.e. 7285 parasites/µL, 10/µL, 2/µL, 1/µL and 0.5/µL).

### Cross-reactivity

No cross-reactivity was observed with rheumatoid factor or with the other blood parasites tested (*Schistosoma*, *Leishmania*, *Trypanosoma*). All 5 samples gave a negative test results on illumigene assay.

## Discussion

The illumigene assay is designed to detect parasites of the genus *Plasmodium* and is developed as a qualitative screening test for malaria infections. An advantage of LAMP assays in comparison to real-time PCR assays is the independence of expensive thermal cyclers. Only a compact incubator/reader is needed. Single testing or batch testing up to 10 samples at the same time is possible. The commercial illumigene assay offers a standardized method, which is easy to use, requiring minimal operator skills. The manufacturer claims a 100.0% sensitivity and 89.3% specificity in comparison with expert microscopy [21]. Another study from the manufacturer claims a 99% sensitivity and 100% specificity compared with microscopy and PCR [22]. The data presented in this study support these findings and are in line with previous published data [25]. In contrast to Lucchi et al. who evaluated the assay in an endemic situation, this study focused on validating the illumigene assay in a non-endemic setting, where infections with low parasite densities are more frequent [26] and urgent diagnosis is essential to optimize treatment and reduce mortality. The illumigene LAMP assay results were compared to microscopy, RDT and real-time PCR. Data on the performance of a LAMP test in comparison to real-time PCR is rare.

In the retrospective experiment the illumigene assay showed 100% agreement with the real-time PCR method performed at the Belgian reference laboratory. Seven samples from patients, recently treated for *P. falciparum* infection, were also retrospectively tested on the platform and yielded, as expected, positive test results. As these samples still contained HRP-2 and *P. falciparum*-specific pLDH antigens they were also positive with RDT and real-time PCR, but no longer showed detectable parasite levels on expert microscopy. In the prospective panel, the performance of the illumigene assay equals that of RDT combined by microscopy, the current diagnostic approach in most laboratories in non-endemic regions. The illumigene assay detected three more positive samples than the BinaxNOW RDT alone and one additional positive sample compared to microscopy only in a patient with a history of multiple malaria infections. The positive LAMP result could not be confirmed with real-time PCR on thin blood smear, possibly due to inferior sample quality. The most plausible reason for the discordance with microscopy in this patient is prior treatment. Based on a dilution series, the LoD of the tested assay was at least 0.5 parasites/µL and agreed to the manufacturer’s claim for *P. falciparum* (i.e. 2 parasites/µL) [21]. In the panel of clinical samples 22 samples with a parasitaemia < 500/µL and 7 with densities < 100/µL were positive with the LAMP test. Molecular testing for *Plasmodium* DNA is clearly of value in cases where microscopy and/or RDT lack sensitivity [14].

The illumigene assay was designed for testing human venous whole blood samples (50 µL) with EDTA as preservative [21, 23]. Simpler sample collection methods such as the use of a finger prick blood and filter papers may be preferable in field settings in resource-limited regions. However, the described evaluation was not designed to address the performance on capillary whole blood. This study was conducted in a non-endemic region without any resource limitations, therefore venous blood was relatively easily obtainable. Moreover, the collection method of capillary blood can be unpractical, the sampling of 50–100 µL of whole blood sometimes cumbersome, and moreover the assay requires a sample anticoagulated with EDTA. Therefore, future studies aimed at evaluating this assay for detection of malaria in fresh capillary blood are warranted. Lucchi et al. [25] describe that they do not see any scientific reason why finger prick whole blood samples would not work with the LAMP assay described.

A shortcoming of the illumigene assay is that species identification and determination of parasite density are not possible with this commercial LAMP assay. The development of species-specific primers for *Plasmodium* species identification, is technically challenging and requires experience in PCR method development. Nevertheless, in-house designed malaria LAMP assays have been described capable of detecting both the *Plasmodium* genus as well as the 5 different human infecting species [14, 27]. The need to identify the infecting species and the parasite density is, especially in non-endemic regions, important in order to provide the correct anti-malarial therapy and in providing valuable information on the burden and relative distribution of malaria species [25]. When using the illumigene LAMP assay, species identification and determination of parasite density in positive samples through microscopy and/or real-time PCR remains necessary.

The results in this study suggest that the illumigene assay can be considered as an alternative first-line diagnostic tool instead of the combined approach of RDT and microscopy in non-endemic settings. The LAMP detected three *Plasmodium* infections that were negative with the BinaxNow RDT: one containing *P. ovale* (59 parasites/µL), one containing *P. vivax* (340 parasites/µL) and one containing *P. falciparum* (415 parasites/µL). This finding is in line with the compiled accuracy data for RDTs, i.e. variable sensitivity for the different species and variable performance dependent on the used RDT brand. In general most RDTs used, have high sensitivity for detecting *P. falciparum* but lower for other *Plasmodium* species [6, 12, 14]. Possible reasons for false-negative RDTs are most frequent low parasite density, and in case of *P. falciparum* prozone effect (rare) [28] and parasites with HRP2-gene deletion (rare) [29, 30].

**Fig. 1:**
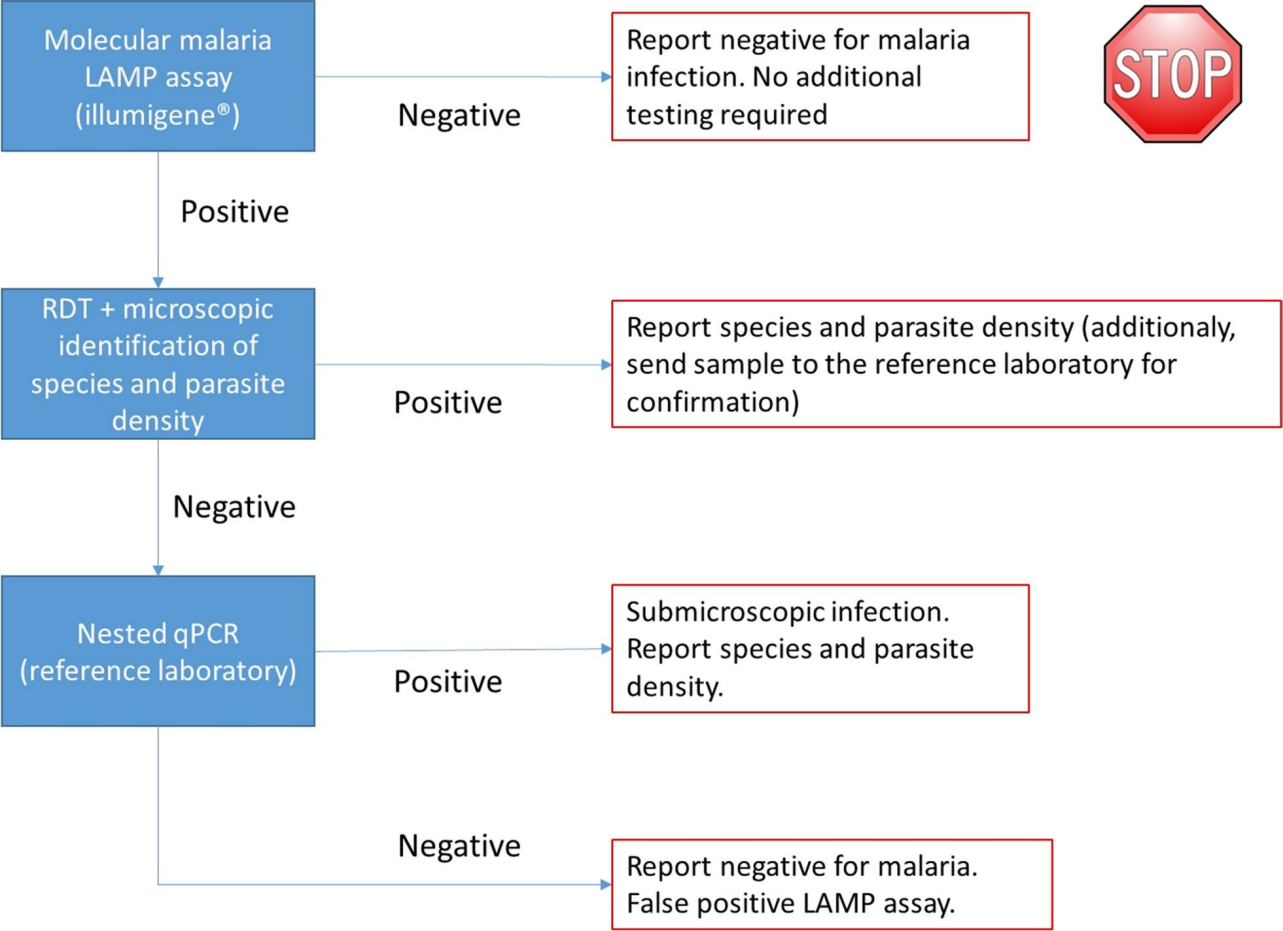
Proposal of a novel diagnostic approach of malaria infections in non-endemic setting. Proposed position of illumigene malaria assay in the diagnostic approach of malaria infections in non-endemic settings with low prevalence. Because of the high sensitivity of the illumigene malaria assay, it seems no longer required to confirm negative results. However, due to low sample size our results alone are inadequate to validate this approach and before implementation, this approach needs to be confirmed in larger, prospective and preferably multi-center studies

Based upon the superior sensitivity and specificity of this assay compared with RDT and microscopy, the following novel approach could be considered in non-endemic settings, particularly in situations with low positivity rates (Fig. 1): implementation of a molecular assay/LAMP assay as a primary diagnostic test. Only positive results would require further diagnostic work-up with microscopy and real-time PCR (reference laboratory), in order to determine parasite density and species identification. Because of the high sensitivity of the illumigene assay, confirmation of negative results by means of microscopy may not be necessary anymore. By using this approach, the majority of samples submitted to the laboratory for suspected clinical malaria infection, no longer requires microscopic evaluation. This approach can be time-saving. However, due to low sample size results are inadequate to fully validate this approach and before implementation, the high sensitivity of this assay needs to be confirmed in larger, prospective and preferably multi-center studies.

A minimum training for laboratory technicians was required to perform the illumigene assay. The sample preparation, incubation of samples and reading of test results is easy to perform as it does not require high-level technical expertise. Hands-on time is limited to less than 10 min and results were available within 1 h. Results are shown as positive, negative or invalid and, therefore, require no subjective interpretation. All reagents are provided in single-use packages and can be preserved for several months (i.e. 12 months, claim from the manufacturer, not tested in this study). Single testing or batch testing up to 10 samples at the same time is possible. This in contrast to real-time PCR where batch testing is required to save costs. During the validation no technical errors nor invalid test results were encountered. However, LAMP tests are currently more expensive than either microscopy and RDT [31]. The cost-effectiveness of the assay will depend on the number of samples and positivity rate, the experience of the microscopists and must be validated in every laboratory independently.

## Conclusion

In conclusion, the excellent performance of the illumigene assay can lead to a fast, more accurate exclusion of clinical suspected malaria infection. These data provide additional support that this assay can be used as initial screening test before RDT and microscopy. Furthermore LAMP assays remain positive after treatment. Larger studies are required to confirm our results. Unfortunately, this LAMP assay cannot identify the *Plasmodium* species nor determine parasite density. Therefore, expert microscopy remains the gold standard method for confirmation of malaria infection.
